# 

**Published:** 1842-06

**Authors:** 


### THE WESTERN JOURNAL OF MEDICINE AND SURGERY:

EDITED BY
DANIEL DRAKE, M. D.
AND
LUNSFORD P. YANDELL, M. D.
PROFESSORS IN THE LOUISVILLE MEDICAL INSTITUTE,
AND
THOMAS W. COLESCOTT, M. D.

NO. XXX.—JUNE, 1842.
VOL. V.—No. VI.

LOUISVILLE, KY.
PUBLISHED MONTHLY, BY PRENTICE & WEISSINGER,
PROPRIETORS AND PRINTERS.
1842.

*This Number contains 4 sheets—Postage under* 100 *miles* 6 *cents, over* 100 *miles* 10 *cents.*

